# Risk factors of post-discharge under-five mortality among Danish children 1997-2016: A register-based study

**DOI:** 10.1371/journal.pone.0226045

**Published:** 2019-12-04

**Authors:** Andreas Jensen, Per Kragh Andersen, John Sahl Andersen, Gorm Greisen, Lone Graff Stensballe

**Affiliations:** 1 Department of Paediatrics and Adolescent Medicine, Rigshospitalet, Copenhagen University Hospital, Copenhagen, Denmark; 2 Section of Biostatistics, Department of Public Health, University of Copenhagen, Copenhagen, Denmark; 3 Section of General Practice and Research Unit for General Practice, Department of Public Health, University of Copenhagen, Copenhagen, Denmark; 4 Department of Neonatology, Rigshospitalet, Copenhagen University Hospital and the University of Copenhagen, Copenhagen, Denmark; University of Hong Kong, CHINA

## Abstract

**Objectives:**

Estimating associations between somatic and socioeconomic risk factors and post-discharge under-five mortality.

**Design:**

Register-based national cohort study using multiple Cox regression.

**Participants:**

The population of 1,263,795 Danish children live-born 1997–2016 who survived until date of first discharge to the home after birth was followed from that date until death, emigration, 5 years of age or 31 December 2016.

**Main outcome measures:**

(A) Mortality hazard ratios (HRs) among all children, (B) mortality HRs among children without severe chronic disease, and (C) mortality HRs among children without severe chronic disease or asthma.

**Main results:**

In the total population (1,947 deaths) severe chronic disease was associated with mortality HR = 15.28 (95% CI: 13.77–16.95). In children without severe chronic-disease (719 deaths) other somatic risk factors were immature birth HR = 3.40 (1.92–6.02), maternal smoking HR = 1.84 (1.55–2.18) and low birth weight HR = 1.74 (1.21–2.51). Socioeconomic risk factors for mortality included: maternal age < 25 years HR = 1.91 (1.38–2.64) compared to > 35 years (similar for 30–35 years and 25–29 years), lowest vs. highest family income tertile HR = 1.76 (1.23–2.51), not living with both parents HR = 1.63 (1.25–2.13), maternal unemployment HR = 1.54 (1.12–2.12), presence of siblings HR = 1.44 (1.20–1.71) and secondary vs. tertiary parental education HR = 1.33 (1.07–1.65) for fathers and HR = 1.23 (1.01–1.52) for mothers. Factors not found to be associated with child mortality in this population included presence of asthma HR = 1.29 (0.83–1.98) and non-Danish ethnicity HR = 0.98 (0.70–1.37).

**Conclusions:**

Childhood death after discharge to the home after birth and before 5 years of age is a very rare event in Denmark. This ‘post-discharge’ mortality was heavily associated with severe chronic disease. In children without severe chronic disease, immature birth, maternal smoking and certain socioeconomic characteristics were noticeable risk factors. Mortality may possibly be decreased by focusing on vulnerable groups.

## Introduction

Under-five mortality has substantially decreased over the years; according to a recent study infant mortality in the Nordic countries was more than halved in the last two decades [1]. However, the study did not examine mortality risk factors in this high-income setting.

Socioeconomic inequalities have been found to persist despite free access to health services [2,3]. Internationally, the associations between economical means, health conditions and under-five mortality have often been subject to investigation [4,5].

Zylbersztejn et al [6] did compare all-cause child mortality in two high-income countries, England and Sweden, and found that the excess mortality in England compared to Sweden was largely attributable to certain characteristics known at birth including gestational age, birth weight, congenital anomalies, maternal age, and socioeconomic status. Partially inspired by these findings Brownell and Enns [7] called for further research on risk factors for child mortality in high-income countries.

Most under-five deaths occur in the neonatal period, and even 25.5% of infant mortality and 14.5% of under-five mortality occurs before first discharge to the home after birth (data under publication). These deaths are likely to be due to the typical neonatal causes such as extreme prematurity, severe birth complications or severe malformation, but these deaths have increasingly often been delayed by active and intensive care to after 28 days of life. As the health care system providing for infants directly after birth (obstetric and neonatal departments) is different from health care institutions that care for infants after discharge (general practice, primary care paediatricians, and paediatric departments and paediatric hospitals) we found interest in focusing on the ‘post-discharge’ mortality as an alternative to the usual post-neonatal mortality which is defined as mortality after 28 days without taking into account any conditions on the specific child. Children who were discharged to the home are more likely to be healthy individuals with a fair chance of survival. Thus, the two mortality definitions target different health care challenges. We previously found that the decrease in this post-discharge mortality during the last 20 years has been even more dramatic than the conventional ‘post-neonatal’ mortality (data under publication).

To judge if the current Danish health care system matches the needs, and is adapted to current risk factors for death, we undertook a new analysis of our dataset–aiming to identify risk factors which may be better addressed by focused preventive measures. We selected and divided risk factors [6,8] in four types depending on their temporal order—before, during and after pregnancy and subsequent child disease—to obtain the appropriate confounder structure for the estimate of each risk factor. For further clarity, each risk factor was labelled as either somatic or socioeconomic. Among somatic risk factors one focus was chronic disease, where we examined the implications of severe, chronic, rare conditions. In addition, we examined asthma, the most frequent chronic disease in childhood, however often with only mild to moderate influence on the child. In the analysis of post-discharge under-five mortality, each risk factor was adjusted for the set of confounders placed before or at the same step in the temporal hierarchy. In addition, crucially, all estimates were adjusted for the calendar time trend of decreasing mortality.

Thus, the aim of the study was to examine risk factors for post-discharge mortality. Knowledge of these factors may lead to health care strategies with the purpose of further reduction of child mortality.

### Healthy children

In the present study we do not define asthma or asthma-like disease as a severe chronic disease. But it is a much more frequent condition affecting 20–30% of our child population [9] and it has been called an epidemic in high-income countries. We aimed at distinguishing between (A) all children, (B) children without severe chronic disease (which we called the ‘healthy’ children), and (C) children with neither severe chronic disease nor asthma. The purpose of the present study was to examine risk factor profiles in these populations. The pathway to health care for healthy children is quite different from that of children with a diagnosis of a severe chronic disease and the challenges for the health care system are also quite different: for the first group it is a large volume/low risk task, while for the last group it is a small volume/high risk task. Therefore, we found it interesting to also separately examine risk factors of mortality in healthy children.

## Methods

The background study population consisted of all live-born Danish children born from 1997 to 2016. In addition, past information on the parents of all live-born Danish children born from 1997 to 2016 was available from two years before birth.

During the study period 1,268,222 children were born alive in Denmark. The follow-up period was defined as the time from the first discharge to the home after birth until the first of the following events: death, emigration, 5-year birthday, 31 December 2016. Children born at home contributed with person-time from the date of birth. Some children either died before discharge (which was the start of follow-up) or remained in hospital until 31 December 2016 (end of follow-up). The outcome analysed was post-discharge mortality, and thus these children were excluded. Children with registered migration between birth and discharge to the home were excluded as well. The final study population consisted of 1,263,795 children.

The data was extracted from the Civil Personal Register [10] covering the entire Danish population, and from the following national registers: the Cause of Death Register [11], the Medical Birth Registry [12], the National Patient Register [13], the Register of Medicinal Product Statistics [14], and various databases containing socioeconomic information [15]–Further details on the registries can be found in the Supporting information (Table A in S1 File).

### Risk factors and statistics

**Fig 1:**
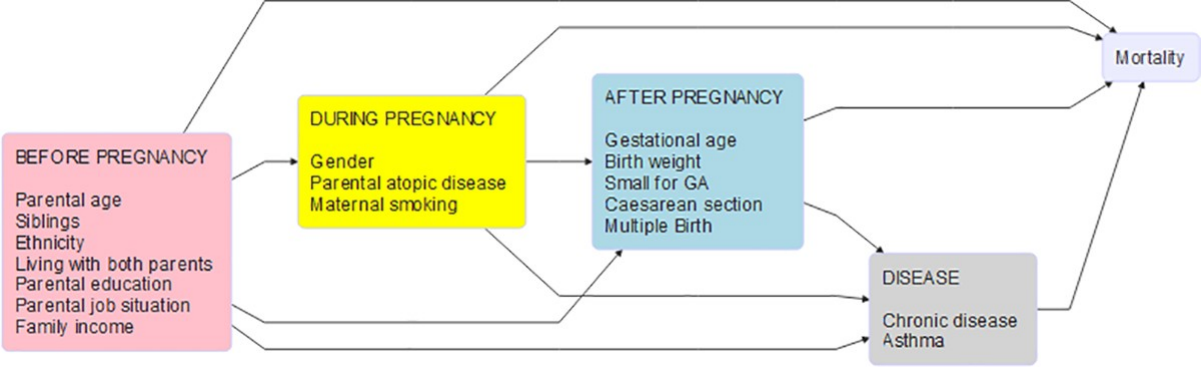
Causal diagram on the mortality risk factor pathways–four set of risk factors and their temporal order. Temporal hierarchy of the risk factor sets. In the analysis of a given risk factor only the set of variables positioned at the same time or earlier in the temporal hierarchy were adjusted for as confounders.

The statistical analyses were set out to explore the associations between various selected risk factors and post-discharge mortality. The first analysis described here is referred to as analysis (A) whereas two other analyses (B) and (C) will be presented later in the methods section under the subheading ‘Risk factors among healthy children’. To guide the confounder selection for each individual risk factor estimation, the risk factors were grouped in four temporal types and classified as either socioeconomic or somatic (see Table 1). This approach resulted in four different classes of models where different sets of risk factors were viewed as the exposure in a classical epidemiological sense. Thus, conditioning on mediating variables was avoided, and it was ensured that only possible causes for the risk factor and for post-discharge mortality were included as confounding variables. Considering variables within the same set as one node in the diagram (cf. Fig 1) was justified by the disjunctive cause criterion (VanderWeele et al [16]).

**Table 1:** Definition of risk factors and the confounder structure of their possible association with post-discharge under-five mortality.

		List of risk factors and their temporal order		
Temporal group	Type	Risk factor	Levels	Set of confounders in the analysis of risk factor
Before pregnancy	Socioeconomic	Maternal age	< 25y, 25-29y, 30-35y, > 35 years	Before pregnancy
		Paternal age	< 25y, 25-29y, 30-35y, > 35 years	
		Siblings	no, yes	
		Ethnicity	Danish, other	
		Living with both parents	no, yes	
		Maternal education	primary, secondary, tertiary	
		Paternal education	primary, secondary, tertiary	
		Maternal job situation	employed, unemployed, out of workforce, student	
		Paternal job situation	employed, unemployed, out of workforce, student	
		Family income*	lowest tercile, mid tercile, highest tercile	
		Birth year	*continuous*	Not of explicit interest but serves to adjust for decreasing child mortality
During pregnancy	Somatic	Gender	female, male	Before and during pregnancy
		Maternal atopic disease [9]	no, yes	
		Paternal atopic disease [9]	no, yes	
		Smoking	no, yes	
After pregnancy	Somatic	Gestational age	< 28w, 28-37w, > 37 weeks	Before, during and after pregnancy. Severe chronic disease and asthma fixed at first discharge to the home after birth
		Birth weight	< 2500 g, ≥ 2500 g	
		Small for gestational age [17]	no, yes	
		Caesarean section	no, yes	
		Multiple birth	no, yes	
Disease	Somatic	Severe chronic disease [18]	absent, present	Time-dependent and adjusted for all other riskfactors
		Asthma [9]	absent, present	

* Family income tercile relative to the distribution within year of birth.

As explained in Table 1, the analysis of each risk factor was adjusted for the set of other risk factors positioned at the same place or earlier in the temporal hierarchy. This effectively meant that four different model structures were identified: 1) one using only before pregnancy information, 2) one using before- and during pregnancy information, 3) one using all information ascertained at discharge, and 4) one also using time-dependent versions of severe chronic disease and asthma (more on this below).

In addition, the analyses were linearly adjusted for year of birth defined as the integer number of years since 1997. The (severe) chronic disease and asthma variables were time-dependent with the status updated at pre-specified ages (28 and 365 days) and on every 1 January using definitions from our prior studies [9,18]. These algorithms were based on register information on prescriptions (asthma) and hospital contacts (asthma and chronic disease). However, the definition of severe chronic disease was slightly modified, and the changes are stated in the Supporting information (Table B in S1 File).

The asthma variable was defined as in the reference [9], but only considering asthma-related prescriptions and hospital contacts and not atopic disease in general (the algorithm used in the reference also takes atopic dermatitis and allergic rhinoconjunctivitis into account).

The baseline risk factors of maternal and paternal atopic disease were defined using the original algorithm in [9] but applied to the parents considering information within two years prior to the birth of the child. Incompletely registered variables were handled by defining a separate category for missing values. The time to death was analysed using Cox regression with age as the underlying time scale, and delayed entry at first discharge to the home after birth. As a sensitivity analysis all the standard errors were corrected analysing siblings as belonging to the same cluster.

### Risk factors among healthy children

The first analysis described in the methods section is referred to as analysis (A)—all children. Two additional analyses (B) and (C) were carried out to restrict attention to the so-called healthy children–i.e. those not suffering from severe and potentially life-threatening chronic disease (B), and those who in addition were not suffering from asthma (C):

(B) In this analysis children with severe chronic disease defined at the hospital before first discharge were excluded. Children diagnosed with a severe chronic disease later in the follow-up period were censored at the first date of diagnosis, and thus the estimated parameters were hazard ratios for death in a population of healthy children.

(C) Follows the same approach as in (B), but with further censoring at dates of asthma diagnoses. Thus, the outcome in this analysis was mortality among children without any severe chronic disease and asthma according to our stated definitions.

Censoring for severe chronic disease/asthma does not affect the consistency of the hazard ratio estimates under the assumption that the censoring mechanism was independent of the mortality hazard given the remaining risk factors in the model. This assumption was more likely to be fulfilled in the estimates corresponding to the after pregnancy-risk factors which were placed at the bottom of the temporal hierarchy explained in Table 1.

### Data quality

Various reviews concluded that Danish national health registers are complete and are valuable tools for epidemiological research [13,19,20]. Using the unique personal identification number of all Danish citizens the registers enable individual-level linkage between various sources of information including contacts with the health system [10]. In the present study the outcome variable, all-cause mortality, is very accurately ascertained, whereas the reliability of cause-specific mortality relies upon the notification of the physicians and the coding of the cause of death variable [11].

## Results

The mortality hazard ratios corresponding to the risk factors are presented in Table 2. The rightmost columns represent the estimates in populations (B) and (C) in which the children with severe chronic disease respectively severe chronic disease *or* asthma were censored. The estimate corresponding to each risk factor was extracted from the corresponding model as explained in Table 1 above. Thus, within each of the three populations (A)-(C) the estimates were obtained from four models with increasing degree of adjustment.

**Table 2:** Baseline distribution of risk factors and their associations with post-discharge under-five mortality. Column (A) represents all eligible children, (B) children without severe chronic disease, (C) children without severe chronic disease *or* asthma.

Under-five mortality hazard ratios (95% CI) corresponding to the risk factors				
Population		*(A) All children*	*(B) Children without severe chronic disease*	*(C) Children without asthma or chronic disease*
Number of children entering the analysis		1,263,795	1,246,599	1,245,998
Number of deaths		1,947	719	690
**RISK FACTORS (ref = reference level)**				
	*Baseline % (N = 1*,*263*,*795)*			
**Socioeconomic risk factors**				
***Maternal age (ref > 35y)***				
< 25 years	12.95%	1.62 (1.33–1.99)	1.91 (1.38–2.64)	1.83 (1.32–2.55)
- 25–29 years	33.28%	1.07 (0.90–1.27)	1.07 (0.80–1.42)	1.05 (0.79–1.41)
- 30–35 years	40.15%	0.94 (0.80–1.09)	0.88 (0.68–1.15)	0.87 (0.66–1.13)
***Paternal age (ref > 35y)***				
< 25 years	6.02%	1.06 (0.86–1.31)	1.30 (0.95–1.80)	1.37 (0.99–1.90)
- 25–29 years	23.70%	0.90 (0.77–1.05)	0.99 (0.77–1.26)	0.98 (0.76–1.26)
- 30–35 years	41.69%	0.93 (0.83–1.06)	0.85 (0.69–1.05)	0.86 (0.69–1.06)
Siblings	53.69%	1.17 (1.05–1.30)	1.44 (1.20–1.71)	1.43 (1.19–1.71)
Non-Danish ethnicity	10.22%	1.24 (1.02–1.49)	0.98 (0.70–1.37)	0.98 (0.69–1.38)
Not living with both parents	10.61%	1.58 (1.35–1.86)	1.63 (1.25–2.13)	1.64 (1.24–2.17)
***Maternal education (ref*: *tertiary)***				
- Primary	0.64%	1.22 (0.79–1.90)	1.16 (0.55–2.44)	1.20 (0.57–2.54)
- Secondary	55.95%	1.18 (1.05–1.33)	1.24 (1.01–1.52)	1.23 (1.00–1.51)
***Paternal education (ref*: *tertiary)***				
- Primary	0.42%	1.00 (0.57–1.76)	1.03 (0.41–2.57)	1.08 (0.43–2.70)
- Secondary	62.36%	1.27 (1.12–1.44)	1.33 (1.07–1.65)	1.37 (1.10–1.71)
***Maternal job situation (ref*: *employed)***				
- Unemployed	3.36%	1.17 (0.94–1.45)	1.54 (1.12–2.12)	1.50 (1.07–2.08)
- Outside workforce	15.21%	0.97 (0.86–1.10)	1.04 (0.86–1.26)	1.06 (0.87–1.29)
- Student	9.57%	0.86 (0.72–1.02)	0.77 (0.58–1.04)	0.79 (0.59–1.07)
***Paternal job situation (ref*: *employed)***				
- Unemployed	2.44%	1.09 (0.86–1.39)	1.14 (0.79–1.66)	1.16 (0.79–1.69)
- Outside workforce	7.63%	0.86 (0.73–1.00)	0.83 (0.64–1.06)	0.82 (0.63–1.05)
- Student	4.86%	0.73 (0.58–0.91)	0.50 (0.33–0.76)	0.52 (0.34–0.78)
***Family income (ref*: *high tertile in year)***				
- lowest tertile in year	by definition	1.31 (1.09–1.57)	1.76 (1.23–2.51)	1.73 (1.19–2.51)
- middle tertile in year	by definition	0.95 (0.79–1.15)	1.33 (0.92–1.90)	1.38 (0.95–2.01)
***Year of birth***	*continuous*	0.90 (0.89–0.91)	0.88 (0.87–0.90)	0.88 (0.87–0.90)
**Somatic risk factors**				
Male sex	48.71%	1.00 (0.91–1.09)	0.97 (0.83–1.12)	0.96 (0.82–1.11)
Maternal atopic disease	4.19%	0.97 (0.80–1.18)	1.00 (0.73–1.38)	0.96 (0.69–1.34)
Paternal atopic disease	3.58%	0.94 (0.74–1.19)	0.84 (0.55–1.29)	0.81 (0.52–1.27)
Maternal smoking	16.29%	1.39 (1.25–1.55)	1.84 (1.55–2.18)	1.84 (1.55–2.20)
***Gestational age (ref >37w)***				
- Premature (28-37w)	6.95%	1.00 (0.84–1.18)	1.00 (0.73–1.37)	1.01 (0.73–1.38)
- Immature (< 28w)	0.21%	1.28 (0.93–1.78)	3.40 (1.92–6.02)	3.36 (1.89–5.97)
Birth weight < 2500g	5.10%	1.44 (1.17–1.77)	1.74 (1.21–2.51)	1.87 (1.29–2.70)
Small for gestational age	3.03%	1.17 (0.97–1.42)	1.21 (0.87–1.70)	1.16 (0.83–1.64)
Caesarean section	19.87%	1.14 (1.02–1.27)	1.24 (1.02–1.50)	1.22 (1.01–1.49)
Multiple birth	3.85%	0.85 (0.69–1.05)	1.29 (0.93–1.80)	1.23 (0.87–1.73)
Severe chronic disease	1.36% ^(*)^ ^time-dependent^	15.28 (13.77–16.95)	*Not defined*	*Not defined*
Asthma	0.06% ^(*)^ ^time-dependent^	1.55 (1.28–1.87)	1.29 (0.83–1.98)	*Not defined*

Note that the estimates corresponding to the respective risk factors are derived from four classes of models with increased degree of adjustment.

(*) During follow-up 4.67% of the children developed severe chronic disease, and 11.05% developed asthma

According to Table 2, severe chronic disease was the most important risk factor for post-discharge death, HR = 15.28 (95% CI: 13.77–16.95), a minority of 1.36% of the children at baseline (eventually increasing to 4.67% during follow-up) accounting for nearly two thirds of the mortality (1-719/1947 = 0.63 i.e. 63%). The role of other risk factors can be assessed by comparing estimates in analysis (A) with the corresponding estimates in analysis (B). For example, the estimate of immature birth (gestational age less than 28 weeks) was amplified from HR = 1.28 (0.93 to 1.78) to HR = 3.40 (1.92–6.02) when censoring children with severe chronic disease. The estimates in analysis (B) and (C)–the latter also censoring children with asthma–were not very different. Thus, the population in (B) was also referred to as the healthy children.

Apart from immature birth the following somatic risk factors were associated with the largest mortality hazard ratios among the healthy children: maternal smoking HR = 1.84 (1.55–2.18), birth weight less than 2.5 kilograms HR = 1.74 (1.21–2.51), and, to a lesser extent, caesarean section HR = 1.24 (1.02–1.50).

The following socioeconomic risk factors were associated with the largest hazard ratios among healthy children: maternal age < 25 was associated with an increased hazard HR = 1.91 (1.38–2.64) compared to maternal age > 35 years (the hazards in the three oldest age groups were mutually similar). Low paternal age also seemed to be associated with increased mortality albeit to a lesser extent., being in the lowest family income tertile HR = 1.76 (1.23–2.51), not living with both parents HR = 1.63 (1.25–2.13), maternal unemployment HR = 1.54 (1.12–2.12) and presence of siblings HR = 1.44 (1.20–1.71) were also associated with increased mortality. In addition, note that children of studying fathers had a lower hazard of mortality HR = 0.50 (0.33–0.76) compared to that of children with fathers in employment. The mortality in children of parents with secondary education was higher than that of children of parents with tertiary education, HR = 1.33 (1.07–1.65) for fathers and HR = 1.24 (1.01–1.52) for mothers (robust 95% CI: 1.00–1.54).

As mentioned, all the estimates were adjusted for birth year assuming a linear decrease in mortality over time. The calendar association itself was an estimated 10% among the healthy children decrease in the mortality hazard per year since 1997.

The sensitivity analysis applying robust standard errors to accommodate the correlation between siblings did not change the conclusions based on the 95% confidence intervals.

## Discussion

### Findings

The present register-based national cohort study investigated somatic and socioeconomic risk factors and their association with under-five post-discharge mortality among live-born Danish children from 1997 to 2016.

In the general population of children, the key finding was the large magnitude of the association between severe chronic disease and under-five mortality. The presence of severe chronic disease increased the hazard of early childhood mortality more than 15-fold. Less than 5% of the children entering the study were eventually diagnosed with severe chronic disease, and yet almost two thirds of the deaths were discarded by censoring these children. This finding is in line with that of a previous study from the United Kingdom which estimated that 65.4% of child mortality was attributable to chronic disease [21].

We also found it interesting to study the impact on early childhood mortality of asthma, the most common chronic disease in childhood. We did not find diagnosis of asthma to be associated with the mortality hazard among the otherwise healthy children. And including asthma diagnoses in the censoring mechanism did not change the remaining risk factor estimates markedly, and only in few cases was the cause of death among children with asthma classified as respiratory disease (Table C in S1 File). Thus, we focus on the results of the analysis of the healthy children (i.e. children without severe chronic disease but possibly with asthma). In this healthy child population few risk factors were associated with large point estimates. In the total study population including children with severe chronic disease, gestational age was not predictive of mortality given the chronic disease status. The pattern was different among healthy children with the mortality hazard of immature children (< 28 weeks of gestation) being more than 3-fold that of children born at term. However, these children constituted a small subgroup (0.21% of the children). In contrast, low birth weight was “only” associated with a doubling of hazard, but as the proportion of children born with low birth weight was 5%, this is more important at the population level.

Maternal smoking was already a well-known risk factor and in the healthy population the estimated mortality hazard was almost twice as high among children of smokers compared to children of non-smokers. Low birth weight was another somatic risk factor with an estimated increased mortality hazard of 74%. Gestational age, birth weight, and weight-for-gestational age are related, and the parameter estimates may be conceived as a prematurity/low birth weight cluster of genetic, embryological, foetal and neonatal factors. Our confounder structure may not fully address the complexities of these relations.

Among the socioeconomic risk factors, children of mothers < 25 years of age had higher mortality compared to children of older mothers. If the finding that children of younger mothers had an increased mortality was due to social differences, then these social differences must be manifested by variables not controlled for in the present study. Another study on childhood deaths from cancer found differing directions of the parental age factor depending on the specific cause of death [22]. Children with older siblings had a higher mortality in line with the findings of a previous study regarding risk factors of infection which again is a risk factor of mortality [23].

Note that there were too few events among children of mothers with primary education level to distinguish this group from those with a higher parental education. The hazard ratio between children of mothers with secondary respectively tertiary education was the weakest among the socioeconomic risk factors. Other studies suggested a maternal educational gradient [3,4]. Our models assumed that the mortality hazard decreased linearly with birth year. An exploratory sensitivity analysis ignoring birth year did result in a stronger estimate corresponding to maternal education. In other words; over time child mortality decreased whereas the maternal education level improved. In the present study low family income, not living with both parents, maternal unemployment and presence of siblings were more strongly associated with higher mortality even after adjusting for calendar time. Again, age, status as a student, educational achievement, and income represent factors that may be difficult to dissect. Another study found no significant association between parental education or income and childhood mortality after cancer [24]. However, this population is not easily compared with the population in the present study.

## Strengths and limitations

The strengths of the study included the population-based national coverage, a large sample size, many events, and small loss to follow-up.

Fixing severe chronic disease status at the baseline timepoint or landmark approaches would allow estimation of (absolute) risks and thus the proportion of child mortality attributable to chronic disease and number needed to treat. This was not possible in the time-dependent definition of severe chronic disease in the analyses in the total child population, which constituted a limitation of the chosen approach. However, our approach with an updated version of the severe chronic disease variable most likely gave rise to less misclassification, and most likely a more precise assessment of the association between chronic disease and under-five mortality.

In the two other analyses, children with severe chronic disease, and children with severe chronic disease and asthma were censored resulting in estimations of cause-specific mortality hazards among healthy children and the ability to separately examine the effect of asthma on early childhood mortality. This approach gave rise to less internal misclassification of the severe chronic disease variable and increased the probability that the identified healthy children were indeed healthy. In addition the asthma variable is prone to a degree of misclassification as demonstrated in our previous validation study [25]. Even though the Danish population is small and considered very well registered in public and health registers, random and non-differential misclassification may be present in the register data the present study was based upon.

In relation to the statistical modelling approach a limitation was the lack of explicit exposure-outcome setup which the criterion for confounder selection described by VanderWeele [16] was based on. The present paper dealt with multiple concomitant exposure variables (referred to as *risk factors*) which complicated the undertaken epidemiological methodology.

## Conclusion

The present population-based large-sample cohort study comprising 20 calendar years in a high-income setting presents a comprehensive analysis of various risk factors of post-discharge under-five mortality all of which adjusted for each other. The study demonstrated that children with severe chronic disease had a vastly increased mortality hazard. Asthma was not associated with early childhood mortality among the otherwise healthy children. In healthy children immature birth, maternal smoking and low birth weight increased the hazard of mortality. Maternal age < 25 years (and to a lesser extent lower paternal age), higher family income, living with both parents, maternal unemployment, presence of siblings and secondary parental education were socioeconomic factors associated with increased mortality.

## Supporting information

S1 File Supporting information. (DOCX)
