# Supplementary material for: Risk factors of post-discharge under-five mortality among Danish children 1997-2016: A register-based study
Source: PLoS One. 2019 Dec 4;14(12):e0226045. doi: 10.1371/journal.pone.0226045 (PMC6892471; doi:10.1371/journal.pone.0226045)
Supplement: S1 File — (DOCX) [file pone.0226045.s001.docx]

**Table A in S1 File. List of the Danish national registers upon which the variables used in the paper were based.**

| **Registry** | **Variable** | **Definition** |
| --- | --- | --- |
| The Central Person Register | Date of birth, migration or death |  |
|  | Gender  Ethnicity | Ethnicity dichotomised to Danish or other using the variable IE_TYPE |
| The National Patient Register | Diagnosis of chronic disease or atopic disease | Derived from the variable C_DIAG |
| The Register of Medicinal Product Statistics | Diagnosis of atopic disease | Derived from the variable ATC |
| The Medical Birth Registry | Gestational age  Small for gestational age  Birth weight | Derived from the variables GESTATIONSALDER_DAGE (gestational age in days) and BARN_VAEGT (birth weight of the child) |
|  | Caesarean section  Multiple birth  Parity  Maternal smoking | Derived from MARKOER_KEJSERSNIT (marker of caesarean section), FLERFOLDSGRAVIDITET (pregnant with more than one child), PARITET (parity), and RYGERSTATUS_MODER (smoking status of the mother) |
| Education Register (UDDA, Statistics Denmark) | Education | Categorised from the variable HFAUDD using the internationally standardised ISCED2011 classification |
| Family Income Register (FAIK, Statistics Denmark) | Family income  Parents living together | Derived from FAMAEKVIVADISP_13 (equated family disposable income) and FM_MARK |
| Workforce Classification Module (AKM, Statistics Denmark) | Job situation | Derived from SOCIO13 |

**Text A in S1 File. Definition of chronic disease.**

The children with chronic disease were to a large extent identified using the algorithm referenced in the main paper (1). However, congenital viral infections (ICD-10 codes P35.0–P35.9) were excluded in the algorithm used for the present study, since the clinical presentation of such infections is very variable. The ICD10-codes marked with the (X) symbol in Table S2 below were added to the original set of chronic diseases, since the author group found these conditions to meet the criteria of chronicity and severity:

**Table B in S1 File. List of original and additional diagnoses defining severe chronic disease status.**

| **ICD10-codes** | **Diagnoses** | **Addition**^a)^ |
| --- | --- | --- |
| C00.0-C96.9 | Malignant neoplasms |  |
| D61.0; D61.3; D61.8-D61.9 | Aplastic anaemias | X |
| D76.2 | Haemophagocytic syndrome, infection-associated | X |
| D80.0-D82.9 | Immunodeficiencies |  |
| E10 | Insulin-dependent diabetes mellitus | X |
| E25 | Adrenogenital disorders | X |
| E70.0-73.0 | Disorders of amino-acid metabolism |  |
| E74.0-E84.9 | Disorders of metabolism and cystic fibrosis |  |
| G12 | Spinal muscular atrophy and related syndromes |  |
| G31.0; G31.8–G31.9; G37.0–G37.9 | Degenerative and demyelinating diseases of nervous system | X |
| G40 | Epilepsy |  |
| G60 | Hereditary and idiopathic neuropathy |  |
| G70.2 | Congenital and developmental myasthenia |  |
| G71.0-G71.3 | Mitochondrial myopathy |  |
| G73.6 | Myopathy in metabolic diseases | X |
| G80 | Cerebral palsy |  |
| G81.1; G82.1; G82.4 | Spastic conditions | X |
| G91 | Hydrocephalus |  |
| G94.1 | Hydrocephalus in neoplastic disease | X |
| I12 | Hypertensive renal disease without renal failure | X |
| I27.1-I27.9 | Pulmonary heart disease |  |
| I30.0-I52.8 | Other forms of heart disease |  |
| J44.8 | Other specified chronic obstructive pulmonary disease | X |
| J84 | Other interstitial pulmonary diseases |  |
| K21 | Gastro-oesophageal reflux disease |  |
| K50.0–K51.9 | Crohn disease [regional enteritis] and ulcerative colitis | X |
| K70.0-K77.8 | Diseases of liver |  |
| K90 | Intestinal malabsorption |  |
| M30.0-M35.9 | Systemic involvement of connective tissue |  |
| N03-N05 | Nephritic syndrome | X |
| N07 | Hereditary nephropathy, not elsewhere classified |  |
| N13 | Obstructive and reflux uropathy |  |
| N18.0-N19.9; N25.0-N27.9 | Chronic kidney disease |  |
| P27 | Chronic respiratory disease originating in the perinatal period |  |
| P57.0-P57.9 | Kernicterus | X |
| P91.0-P91.2 | Disturbances of cerebral status of newborn | X |
| P94.0-P94.9 | Disorders of muscle tone of newborn |  |
| Q01-Q07 | Congenital malformations of the nervous system |  |
| Q20.0-Q26.9 | Congenital malformations of the circulatory system |  |
| Q30.0-Q32.4 | Congenital malformations of nose, larynx, trachea and bronchus |  |
| Q34.0-Q37.9 | Other congenital malformations of respiratory system, cleft lip and cleft palate |  |
| Q39.0-Q45.3 | Congenital malformations of upper alimentary tract, intestine and pancreas |  |
| Q60.0-Q64.9 | Congenital malformations of the urinary system |  |
| Q79.0 | Congenital diaphragmatic hernia |  |
| Q79.2-Q79.3 | Exomphalos and gastroschisis |  |
| Q86.0 | Fetal alcohol syndrome (dysmorphic) |  |
| Q87 | Other specified congenital malformation syndromes affecting multiple systems | X |
| Q90.0-Q99.9 | Chromosomal abnormalities, not elsewhere classified |  |

a) The X symbol in the rightmost column indicates an addition to the original list of chronic diagnoses from (1)

**Text B in S1 File. Causes of death.**

Table S3 presents the distribution of deaths by causes as defined in Yu et al (2016) (2). The number of deaths and percentages are shown for the three populations, respectively.

**Table C in S1 File. Number of deaths by different causes in the populations defined by presence of severe chronic disease and asthma.**

| Post-discharge under-five mortality among liveborn Danish children 1997-2016 | **Number of deaths by population**  **(% of all deaths in parenthesis)** | | |
| --- | --- | --- | --- |
| ***Cause of death*** | *All children* | *Children without severe chronic disease* | *Children without asthma and chronic disease* |
| Infectious and parasitic disease | 106 (5.44) | 76 (10.57) | 73 (10.58) |
| Neoplasms | 146 (7.50) | 4 (0.56) | 4 (0.58) |
| Endocrine, nutritional and metabolic diseases | 106 (5.44) | 11 (1.53) | 11 (1.59) |
| Diseases of nervous system | 130 (6.68) | 25 (3.48) | 25 (3.62) |
| Diseases of circulatory system | 49 (2.52) | 14 (1.95) | 14 (2.03) |
| Diseases of respiratory system | 79 (4.06) | 43 (5.98) | 40 (5.80) |
| Diseases of the digestive system | 23 (1.18) | 9 (1.25) | 9 (1.30) |
| Certain conditions originating in the perinatal period | 143 (7.34) | 65 (9.04) | 64 (9.28) |
| Congenital malformations | 538 (27.63) | 40 (5.56) | 39 (5.65) |
| Sudden infant death syndrome | 179 (9.19) | 145 (20.17) | 144 (20.87) |
| Unknown or unspecified causes and other diseases | 158 (8.12) | 59 (8.21) | 57 (8.26) |
| Unnatural death from external causes of injury and poisoning | 245 (12.58) | 201 (27.96) | 184 (26.67) |
| Missing | 45 (2.31) | 27 (3.76) | 26 (3.77) |
| **Total** | 1,947 (100) | 719 (100) | 690 (100) |

**Appendix A in S1 File. Supporting references.**

1. Kristensen K, Hjuler T, Ravn H, Simoes EAF, Stensballe LG. Chronic diseases, chromosomal abnormalities, and congenital malformations as risk factors for respiratory syncytial virus hospitalization: a population-based cohort study. Clin Infect Dis. 2012;54(6):810–7.

2. Yu Y, Qin G, Cnattingius S, Gissler M, Olsen J, Zhao N, et al. Mortality in children aged 0-9 years: A nationwide cohort study from three nordic countries. PLoS One. 2016;11(1).
